# Comparison of inherited retinal disease genes covered by two comprehensive genetic testing panels and a widely used online resource

**DOI:** 10.1038/s41433-025-03629-0

**Published:** 2025-01-28

**Authors:** Asha Vanzara, Elena Schiff, Siying Lin, Jose S. Pulido, Michel Michaelides, Andrew R. Webster, Omar A. Mahroo

**Affiliations:** 1 UCL Institute of Ophthalmology, University College London, London, UK; 2 University of Cambridge, Cambridge, UK; 3 NIHR Biomedical Research Centre at Moorfields Eye Hospital and the UCL Institute of Ophthalmology, London, UK; 4 Manchester Centre for Genomic Medicine, Saint Mary’s Hospital, Manchester University NHS Foundation Trust, Manchester, UK; 5 Translational Ophthalmology, Wills Eye Hospital, Philadelphia, PA USA; 6 Section of Ophthalmology, King’s College London, St Thomas’ Hospital Campus, London, UK

**Keywords:** Disease genetics, Hereditary eye disease

Over 250 genes are associated with inherited retinal diseases (IRDs). Genetic testing increasingly employs targeted gene panels, exome or genome sequencing. Within the UK National Health Service (NHS) Genomic Medicine Service, IRDs are now investigated using whole genome sequencing (WGS) with virtual gene panel analysis (approved genes with diagnostic level evidence) [1]. Many targeted panel tests are also available internationally [2]. One of the largest vision-related panels is the Molecular Vision Laboratory (MVL) “Vision Panel” (>1100 genes) [3]. IRD gene lists are also available online: one of the most frequently consulted resources is “RetNet” (Foundation Fighting Blindness) [4]. We explored the extent of overlap between these panels/lists.

Gene lists were compiled from the above sources. For the NHS Genomic Medicine Service, these were approved (“green”) genes for retinal disorders in the Genomics England PanelApp Version 7.0 [5]. Manual curation removed duplicate or alternative gene symbols, finalizing lists using gene symbols approved by the Human Genome Organization (HUGO) Gene Nomenclature Committee (HGNC). Mitochondrial genes were excluded as were loci where the causative gene has yet to be identified.

After exclusions, total numbers were 278, 313 and 1197 for PanelApp, RetNet and MVL (Version 21.2) respectively (Fig. 1). Of PanelApp genes, 260 (94%) were also found within MVL. Of RetNet genes, 299 (96%) were also found within MVL. Of PanelApp genes, 228 (82%) were also found within RetNet (corresponding to 73% of RetNet genes). 221 genes were common to all lists (79%, 71% and 18% of PanelApp, RetNet and MVL respectively). Eleven genes (4% of PanelApp genes) were unique to PanelApp; 7 genes (2% of RetNet genes) were unique to RetNet; 859 genes (72% of MVL genes) were only found in the MVL list. Supplementary Table 1 lists the curated gene sets, as well as those genes overlapping or unique to each.

**Fig. 1:**
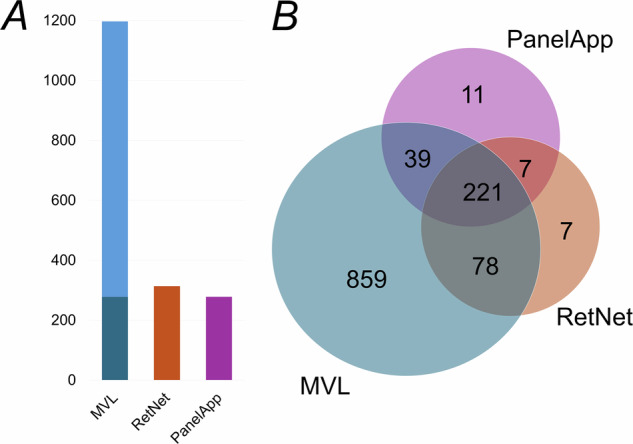
Comparison of genes included in the 3 sources. **A** Total numbers of genes in each list following curation (exclusion of duplicate or alternative non-approved gene symbols, exclusion of mitochondrial genes, exclusion of loci with causative gene not yet identified). For the MVL (Molecular Vision Laboratory) list, the darker blue portion of the bar denotes those genes also designated “retinal dystrophy” genes on the MVL website (although the full panel interrogates all >1000 genes). **B** Venn diagram showing extent of overlap. MVL refers to Molecular Vision Laboratory (MVL) “Vision Panel”, Version 21.2 (accessed 8 Dec 2024). PanelApp refers to approved (“green”) genes for retinal disorders in the Genomics England PanelApp Version 7.0 (accessed 8 Dec 2024). RetNet refers to the genes listed in the online “Retinal Information Network” resource (https://retnet.org/ accessed 8 Dec 2024), provided by the Foundation Fighting Blindness, developed by Dr Stephen P. Daiger with ongoing curation by Dr Lori S. Sullivan at the University of Texas-Houston Health Science Center. Curated gene lists are given in Supplementary Tables 1 and 2.

The MVL website also designates some genes specifically as “retinal dystrophy” genes (although the full Vision Panel interrogates all >1000 genes). After excluding mitochondrial genes, 278 genes are thus listed. We conducted the above analyses also using this list; Supplementary Table 2 gives the curated lists following this analysis, for reference.

Coverage of IRD genes varied between panels/lists, with some genes unique to each list. Over 90% of genes in PanelApp and over 90% of genes in RetNet were covered within the full MVL list. Genes unique to the MVL list partly reflect inclusion of non-IRD eye disease genes. It is important for clinicians to be aware of coverage (and omissions) by gene panels when investigating their patients.

Some limitations should be noted. These findings apply to current versions; optic neuropathy genes fall under a different NHS panel. Read depth will differ by technique. WGS has the advantage of covering intronic regions though variants in such regions might not be routinely analysed. Overall, whether variants are declared, even for included genes, depends on classification pipelines. Panels are continuously evolving, with new genes being added; for PanelApp, efforts are also made to actively downgrade those genes for which evidence for IRD association has weakened.

## Supplementary information

Supplemental Table 1

Supplemental Table 2

## Data Availability

The data processed for the analysis in this manuscript are available on request.
