# Supplementary material for: Comparison of inherited retinal disease genes covered by two comprehensive genetic testing panels and a widely used online resource
Source: Eye (Lond). 2025 Jan 28;39(5):1009–10. doi: 10.1038/s41433-025-03629-0 (PMC11933294; doi:10.1038/s41433-025-03629-0)
Supplement: Supplementary file 1 — Supplemental Table 1 [file 41433_2025_3629_MOESM1_ESM.docx]

**Supplementary Table 1. Genes in each list, unique to each list, or overlapping between lists.** Genes in each list are following curation (exclusion of duplicate or alternative non-approved gene symbols, exclusion of mitochondrial genes, exclusion of loci with gene not yet identified). MVL refers to Molecular Vision Laboratory (MVL) “Vision Panel”, Version 21.2 (<https://www.molecularvisionlab.com/mvl-vision-panel/> accessed 8 Dec 2024). PanelApp refers to approved (“green”) genes for retinal disorders in the Genomics England PanelApp Version 7.0 ( <https://panelapp.genomicsengland.co.uk/panels/307/> accessed 8 Dec 2024). RetNet refers to the genes listed in the online “Retinal Information Network” resource (<https://retnet.org/> accessed 8 Dec 2024).

| **Total for each curated list** | | | **Genes unique to each list** | | | **Genes common to 2 lists only** | | | **Genes common to all 3 lists**  (n=221) |
| --- | --- | --- | --- | --- | --- | --- | --- | --- | --- |
| MVL  (n=1197) | RetNet (n=313) | PanelApp (n=278) | MVL  (n=859) | RetNet  (n=7) | PanelApp  (n=11) | Genes in both MVL and RetNet, but not in PanelApp  (n=78) | Genes in both MVL and PanelApp, but not in RetNet  (n=39) | Genes in PanelApp and RetNet, but not in MVL  (n=7) |  |
| *AARS2*  *AASS*  *ABAT*  *ABCA1*  *ABCA3*  *ABCA4*  *ABCB6*  *ABCB7*  *ABCC6*  *ABCD1*  *ABHD12*  *ACACA*  *ACACB*  *ACAD9*  *ACADL*  *ACADM*  *ACADS*  *ACADVL*  *ACAT1*  *ACBD5*  *ACO2*  *ADAM9*  *ADAMTS10*  *ADAMTS17*  *ADAMTS18*  *ADAMTS2*  *ADAMTSL4*  *ADGRA3*  *ADGRV1*  *ADIPOR1*  *AFAP1*  *AFG3L2*  *AGBL5*  *AGK*  *AGPAT1*  *AGPS*  *AGRN*  *AHI1*  *AHR*  *AIFM1*  *AIPL1*  *AKAP14*  *AKR1C1*  *ALAS2*  *ALDH18A1*  *ALDH1A3*  *ALDH3A2*  *ALG1*  *ALG14*  *ALG2*  *ALMS1*  *ALPK1*  *ALPL*  *AMACR*  *ANGPT1*  *ANKS6*  *ANO10*  *ANTXR1*  *AP3B1*  *AP3D1*  *AP5Z1*  *APC*  *APOB*  *APTX*  *ARFGAP2*  *ARHGEF12*  *ARHGEF18*  *ARID1A*  *ARL13B*  *ARL2*  *ARL2BP*  *ARL3*  *ARL6*  *ARMC9*  *ARMS2*  *ARSG*  *ASB10*  *ASIC5*  *ASPH*  *ASRGL1*  *ATAD3A*  *ATF6*  *ATOH1*  *ATOH7*  *ATP13A2*  *ATP1A3*  *ATP5F1A*  *ATP5F1D*  *ATP5F1E*  *ATP5MF*  *ATP5MG*  *ATP5MGL*  *ATP5PB*  *ATP5PD*  *ATP7B*  *ATPAF1*  *ATPAF2*  *ATXN2*  *ATXN7*  *AUH*  *B3GALNT2*  *B3GALT6*  *B3GLCT*  *B4GALT7*  *B4GAT1*  *B9D1*  *B9D2*  *BBIP1*  *BBS1*  *BBS10*  *BBS12*  *BBS2*  *BBS4*  *BBS5*  *BBS7*  *BBS9*  *BCKDHA*  *BCKDHB*  *BCO1*  *BCO2*  *BCOR*  *BCS1L*  *BEST1*  *BFSP1*  *BFSP2*  *BLOC1S3*  *BLOC1S5*  *BLOC1S6*  *BMP4*  *BMP7*  *BOLA3*  *BTD*  *BUB1B*  *C12ORF57*  *MTRFR*  *C19ORF12*  *C1QBP*  *C1QTNF5*  *C1R*  *C2*  *C2CD3*  *C3*  *INTS15*  *C8A*  *C8B*  *CFAP418*  *C9*  *CA4*  *CA5A*  *CABP4*  *CACNA1A*  *CACNA1F*  *CACNA2D4*  *CANT1*  *CAPN15*  *CAPN5*  *CARS2*  *CASK*  *CAV1*  *CAV2*  *CBS*  *CC2D2A*  *DNAAF19*  *ODAD1*  *CCDC28B*  *CCDC39*  *CCDC40*  *CCER1*  *CCND1*  *CCNP*  *CCT2*  *CD36*  *CDH23*  *CDH3*  *CDHR1*  *CDK5RAP2*  *CDKN2A*  *CDKN2B-AS1*  *CEP104*  *CEP120*  *CEP164*  *CEP19*  *CEP250*  *CEP290*  *CEP41*  *CEP78*  *CEP83*  *CERKL*  *CFAP410*  *CFB*  *CFH*  *CFHR1*  *CFHR2*  *CFHR3*  *CFI*  *CHAT*  *CHCHD10*  *CHD7*  *CHD8*  *CHERP*  *CHKB*  *CHM*  *CHMP4B*  *CHN1*  *CHRNA1*  *CHRNB1*  *CHRND*  *CHRNE*  *CHST14*  *CHST6*  *CIB2*  *CISD2*  *CLCC1*  *CLDN19*  *CLEC3B*  *CLN3*  *CLN5*  *CLN6*  *CLN8*  *CLPB*  *CLPP*  *CLRN1*  *CLTA*  *CLUAP1*  *CNGA1*  *CNGA3*  *CNGB1*  *CNGB3*  *CNNM4*  *CNTNAP2*  *COA3*  *COA4*  *COA5*  *COA6*  *COA7*  *COA8*  *COG4*  *COG6*  *COL11A1*  *COL11A2*  *COL12A1*  *COL13A1*  *COL18A1*  *COL1A1*  *COL1A2*  *COL26A1*  *COL2A1*  *COL3A1*  *COL4A1*  *COL4A2*  *COL4A3*  *COL4A4*  *COL4A5*  *COL5A1*  *COL5A2*  *COL8A1*  *COL8A2*  *COL9A1*  *COL9A2*  *COL9A3*  *COLQ*  *COQ2*  *COQ4*  *COQ5*  *COQ6*  *COQ7*  *COQ8A*  *COQ8B*  *COQ9*  *COX10*  *COX11*  *COX14*  *COX15*  *COX16*  *COX17*  *COX18*  *COX19*  *COX20*  *COX4I1*  *COX4I2*  *COX6A1*  *COX6A2*  *COX6B1*  *COX6B2*  *COX7A1*  *COX7B*  *COX8A*  *CPAMD8*  *CPLANE1*  *CPT1A*  *CPT2*  *CRB1*  *CREBBP*  *CRELD1*  *CRPPA*  *CRX*  *CRYAA*  *CRYAB*  *CRYBA1*  *CRYBA2*  *CRYBA4*  *CRYBB1*  *CRYBB2*  *CRYBB3*  *CRYGA*  *CRYGB*  *CRYGC*  *CRYGD*  *CRYGS*  *CSMD1*  *CSMD2*  *CSPP1*  *CST3*  *CTDP1*  *CTNNA1*  *CTNNB1*  *CTNND1*  *CTSD*  *CTSF*  *CWC27*  *CX3CR1*  *CYC1*  *CYCS*  *CYP1B1*  *CYP27A1*  *CYP4V2*  *CYP51A1*  *DAG1*  *DARS2*  *DBT*  *DCC*  *DCDC1*  *DCN*  *DCT*  *RIGI*  *DGUOK*  *DHCR7*  *DHDDS*  *DHX32*  *DHX38*  *DLAT*  *DLD*  *DMD*  *DNA2*  *DNAAF1*  *DNAAF2*  *DNAAF3*  *DNAAF5*  *DNAH11*  *DNAH5*  *DNAI1*  *DNAI2*  *DNAJC17*  *DNAJC19*  *DNAJC30*  *DNAJC5*  *DNAL1*  *DNM1L*  *DNM2*  *DOCK6*  *DOK7*  *DPAGT1*  *DRAM2*  *DSCAML1*  *DSE*  *DTHD1*  *DTNBP1*  *DYNC2H1*  *EARS2*  *ECHS1*  *EDN3*  *EDNRB*  *EFEMP1*  *ELAC2*  *ELOVL1*  *ELOVL4*  *ELP1*  *ELP4*  *EMC1*  *ENSA*  *EPG5*  *EPHA2*  *ERAL1*  *ERCC1*  *ERCC2*  *ERCC5*  *ERCC6*  *ERCC8*  *ESCO2*  *ESPN*  *ETFA*  *ETFB*  *ETFDH*  *ETHE1*  *EVA1A*  *EVC*  *EVC2*  *EXO5*  *EXOSC2*  *EYA1*  *EYS*  *FA2H*  *FAM131A*  *FAM161A*  *FARS2*  *FASTKD2*  *FBLN5*  *FBN1*  *FBN2*  *FBP1*  *FBXL4*  *FCN1*  *FDFT1*  *FDX2*  *FDXR*  *FERMT1*  *FGF21*  *FH*  *FKBP14*  *FKRP*  *FKTN*  *FLAD1*  *FLNB*  *FLVCR1*  *FOXC1*  *FOXC2*  *FOXE3*  *FOXH1*  *FOXRED1*  *FRAS1*  *FREM2*  *FRMD7*  *FSCN2*  *FTL*  *FXN*  *FYCO1*  *FZD4*  *FZD5*  *G6PC1*  *GAA*  *GALE*  *GALK1*  *GALM*  *GALT*  *GAMT*  *GAN*  *GARS1*  *GAS7*  *GATB*  *GATC*  *GATM*  *GCDH*  *GCNT2*  *GDAP1*  *GDF1*  *GDF3*  *GDF6*  *GFER*  *GFM1*  *GFM2*  *GFPT1*  *GJA1*  *GJA3*  *GJA8*  *GJB1*  *GJB2*  *GJB6*  *GJC3*  *GLA*  *GLIS2*  *GLRX5*  *GMDS*  *GMPPB*  *GNAT1*  *GNAT2*  *GNB3*  *GNPAT*  *GNPTG*  *GNS*  *GPR143*  *GPR160*  *GPR179*  *GPR45*  *GRIP1*  *GRK1*  *GRM6*  *GRN*  *GRWD1*  *GTPBP3*  *GUCA1A*  *GUCA1B*  *GUCA1C*  *GUCY2D*  *GYS2*  *GZF1*  *HADH*  *HADHA*  *HADHB*  *HARS1*  *HARS2*  *HCCS*  *HCN1*  *HESX1*  *HGF*  *HGSNAT*  *HIBCH*  *HK1*  *HKDC1*  *HLCS*  *HMCN1*  *HMGB3*  *HMGCL*  *HMGCS2*  *HMX1*  *HPS1*  *HPS3*  *HPS4*  *HPS5*  *HPS6*  *HSD17B10*  *HSF4*  *HSPD1*  *HTRA1*  *HTRA2*  *HYCC1*  *HYLS1*  *IARS1*  *IARS2*  *IBA57*  *IDH3A*  *IDH3B*  *IDUA*  *IFT122*  *IFT140*  *IFT172*  *IFT27*  *IFT43*  *IFT52*  *TRAF3IP1*  *IFT74*  *IFT80*  *IFT81*  *IFT88*  *IMPDH1*  *IMPG1*  *IMPG2*  *INPP5B*  *INPP5E*  *INTS1*  *INVS*  *IQCB1*  *ISCA1*  *ISCA2*  *ISCU*  *ITM2B*  *JAG1*  *JAM3*  *KARS1*  *KCNJ13*  *KCNV2*  *KCTD7*  *KERA*  *KATNIP*  *KIAA0586*  *KIAA1549*  *KIF11*  *KIF1A*  *KIF24*  *KIF3B*  *KIF7*  *KIT*  *KIZ*  *KLC2*  *KLHL21*  *KLHL7*  *LAMA1*  *LAMA5*  *LAMB1*  *LAMB2*  *LAMP2*  *LARGE1*  *LARS1*  *LARS2*  *LCA5*  *LCT*  *LDLR*  *LEFTY2*  *LEMD2*  *LENG8*  *LHX2*  *LIAS*  *LIM2*  *LIPT1*  *LIPT2*  *LMX1B*  *LONP1*  *LOXL1*  *LOXL3*  *LRAT*  *LRIT3*  *LRMDA*  *LRP2*  *LRP4*  *LRP5*  *LRPPRC*  *LRRC32*  *DNAAF11*  *LSS*  *LTBP2*  *LTBP3*  *LYRM4*  *LYRM7*  *LYST*  *LZTFL1*  *MAB21L1*  *MAB21L2*  *MACF1*  *MAF*  *MAK*  *MAN1C1*  *MAN2B1*  *MAP6D1*  *MAPKAPK3*  *MAPKBP1*  *MARS2*  *MASP1*  *MASP2*  *MC1R*  *MCAT*  *MDH2*  *MECR*  *MEIS1*  *MERTK*  *MFF*  *MFN2*  *MFRP*  *MFSD8*  *MGME1*  *MICOS13*  *MICU1*  *MIEF1*  *MIP*  *MIPEP*  *MIR184*  *MIR204*  *MITF*  *MKKS*  *MKS1*  *MLPH*  *MMACHC*  *MMP1*  *MMP9*  *MORC2*  *MPC1*  *MPRIP*  *MPV17*  *MRM2*  *MRPL12*  *MRPL3*  *MRPL44*  *MRPS14*  *MRPS16*  *MRPS2*  *MRPS22*  *MRPS23*  *MRPS34*  *MRPS7*  *MRRF*  *MRS2*  *MSRA*  *MSTO1*  *MTFMT*  *MTMR7*  *MTO1*  *MTPAP*  *MTTP*  *MUSK*  *MVK*  *MXRA8*  *MYH9*  *MYO5A*  *MYO7A*  *MYO9A*  *MYOC*  *NAA10*  *NADK2*  *NARS2*  *NAXE*  *NBAS*  *NDP*  *NDRG4*  *NDUFA1*  *NDUFA10*  *NDUFA11*  *NDUFA12*  *NDUFA13*  *NDUFA2*  *NDUFA4*  *NDUFA6*  *NDUFA7*  *NDUFA8*  *NDUFA9*  *NDUFAF1*  *NDUFAF2*  *NDUFAF3*  *NDUFAF4*  *NDUFAF5*  *NDUFAF6*  *NDUFAF7*  *NDUFAF8*  *NDUFB11*  *NDUFB3*  *NDUFB6*  *NDUFB8*  *NDUFB9*  *NDUFS1*  *NDUFS2*  *NDUFS3*  *NDUFS4*  *NDUFS5*  *NDUFS6*  *NDUFS7*  *NDUFS8*  *NDUFV1*  *NDUFV2*  *NDUFV3*  *NECTIN2*  *NECTIN3*  *NEFH*  *NEK1*  *NEK2*  *NEK8*  *NEUROD1*  *NF2*  *NFS1*  *NFU1*  *NHS*  *NIPAL3*  *NKX2-5*  *NME8*  *NMNAT1*  *NOD2*  *NODAL*  *NOS3*  *NOTCH2*  *NOTCH3*  *NPHP1*  *NPHP3*  *NPHP4*  *NR2E3*  *NR2F1*  *NRCAM*  *NRL*  *NSUN3*  *NTF4*  *NUBPL*  *NUTF2*  *NXNL1*  *NYX*  *OAT*  *OCA2*  *OCRL*  *OFD1*  *OPA1*  *OPA3*  *OPN1LW*  *OPN1MW*  *OPN1SW*  *OPTC*  *OPTN*  *OR2W3*  *OTC*  *OTOGL*  *OTX2*  *OXA1L*  *P3H2*  *PANK2*  *PARS2*  *PAX2*  *PAX3*  *PAX6*  *PC*  *PCARE*  *PCCA*  *PCCB*  *PCDH15*  *PCK2*  *PCLO*  *PCYT1A*  *PDE6A*  *PDE6B*  *PDE6C*  *PDE6D*  *PDE6G*  *PDE6H*  *PDHA1*  *PDHB*  *PDHX*  *PDP1*  *PDSS1*  *PDSS2*  *PDX1*  *PDXK*  *PDZD7*  *PEAK1*  *PET100*  *PET117*  *PEX1*  *PEX10*  *PEX11B*  *PEX12*  *PEX13*  *PEX14*  *PEX16*  *PEX19*  *PEX2*  *PEX26*  *PEX3*  *PEX5*  *PEX6*  *PEX7*  *PGK1*  *PHYH*  *PIBF1*  *PIK3R1*  *PITPNM3*  *PITRM1*  *PITX2*  *PITX3*  *PKM*  *PLA2G5*  *PLA2G6*  *PLD1*  *PLD3*  *PLEC*  *PLK4*  *PLOD1*  *PLOD3*  *PMP22*  *PMPCA*  *PMPCB*  *PNPLA2*  *PNPLA6*  *PNPLA8*  *PNPT1*  *POC1B*  *POC5*  *POLG*  *POLG2*  *POLRMT*  *POMGNT1*  *POMGNT2*  *POMK*  *POMP*  *POMT1*  *POMT2*  *PON2*  *PORCN*  *PPA2*  *PPT1*  *PRCD*  *PRDM13*  *PRDM5*  *PREPL*  *PRICKLE3*  *PRKCI*  *PROM1*  *PROS1*  *PROX1*  *PRPF3*  *PRPF31*  *PRPF4*  *PRPF6*  *PRPF8*  *PRPH2*  *PRPS1*  *PRR12*  *PRSS56*  *PRX*  *PTCD1*  *PTCD3*  *PTCH1*  *PTPRU*  *PUS1*  *PXDN*  *PYGM*  *QARS1*  *QRSL1*  *RAB18*  *RAB27A*  *RAB28*  *RAB3GAP1*  *RAB3GAP2*  *RAPSN*  *RARB*  *RARS1*  *RARS2*  *RAX*  *RAX2*  *RB1*  *RBMS3*  *RBP3*  *RBP4*  *RCBTB1*  *RD3*  *RDH11*  *RDH12*  *RDH5*  *RECQL4*  *REEP1*  *REEP6*  *RERE*  *RGR*  *RGS6*  *RGS9*  *RGS9BP*  *RHEX*  *RHO*  *RHOBTB2*  *RIMS1*  *RIMS2*  *RLBP1*  *RMND1*  *RNASEH1*  *RNLS*  *ROM1*  *RP1*  *RP1L1*  *RP2*  *RP9*  *RPE65*  *RPGR*  *RPGRIP1*  *RPGRIP1L*  *RPH3A*  *RPL10*  *RPL15*  *RPS19*  *RRAGA*  *RRM2B*  *RS1*  *RSPH4A*  *RSPH9*  *RTBDN*  *RTN4IP1*  *RYR1*  *SACS*  *SAG*  *SALL2*  *SAMD11*  *SARS2*  *SBF2*  *SC5D*  *SCAPER*  *SCARF2*  *SCLT1*  *SCN4A*  *SCO1*  *SCO2*  *SDCCAG8*  *SDHA*  *SDHAF1*  *SDHAF2*  *SDHAF3*  *SDHAF4*  *SDHB*  *SDHC*  *SDHD*  *SEC23A*  *SEMA3A*  *SEMA4A*  *SEMA6A*  *SERAC1*  *SETX*  *SFXN4*  *SH3PXD2B*  *SHH*  *SIL1*  *SIPA1L3*  *SIX1*  *SIX3*  *SIX5*  *SIX6*  *SLC16A12*  *SLC18A3*  *SLC19A2*  *SLC19A3*  *SLC1A5*  *SLC22A5*  *SLC24A1*  *SLC24A5*  *SLC25A1*  *SLC25A12*  *SLC25A13*  *SLC25A15*  *SLC25A19*  *SLC25A20*  *SLC25A21*  *SLC25A26*  *SLC25A3*  *SLC25A32*  *SLC25A33*  *SLC25A38*  *SLC25A4*  *SLC25A40*  *SLC25A42*  *SLC25A46*  *SLC2A1*  *SLC33A1*  *SLC37A4*  *SLC38A8*  *SLC39A13*  *SLC44A1*  *SLC45A2*  *SLC4A4*  *SLC4A7*  *SLC52A2*  *SLC5A7*  *SLC6A15*  *SLC6A8*  *SLC7A14*  *SMOC1*  *SNAI2*  *SNAP25*  *SNRNP200*  *SNX10*  *SNX3*  *SOD1*  *SOX1*  *SOX10*  *SOX2*  *SOX3*  *SPAST*  *AFG2A*  *SPATA7*  *SPG11*  *PLP1*  *NT5C2*  *GBA2*  *DDHD2*  *TFG*  *SPG7*  *SPINT2*  *SPP2*  *SRD5A3*  *SREBF2*  *SSBP1*  *STARD9*  *STAT2*  *STEAP1*  *STEAP2*  *STK38L*  *STRA6*  *SUCLA2*  *SUCLG1*  *SUFU*  *SUOX*  *SURF1*  *SYNE2*  *SYT2*  *SYTL4*  *TACO1*  *TACR1*  *TARS2*  *WWTR1*  *TBC1D20*  *TBC1D32*  *TBCE*  *TBK1*  *TCTN1*  *TCTN2*  *TCTN3*  *TDRD7*  *TEAD1*  *TEK*  *TENM3*  *TFAM*  *TFAP2A*  *TFB1M*  *TGFBI*  *THBS1*  *TIMM22*  *TIMM50*  *TIMM8A*  *TIMMDC1*  *TIMP3*  *TK2*  *TKFC*  *TLCD3B*  *TLR3*  *TLR4*  *TMCO1*  *TMED3*  *TMED7*  *TMEM107*  *TMEM114*  *TMEM126A*  *TMEM126B*  *TLCD5*  *TMEM138*  *TMEM216*  *TMEM218*  *TMEM231*  *TMEM237*  *TMEM65*  *TMEM67*  *TMEM70*  *TMEM98*  *TNPO1*  *TNXB*  *TOP3A*  *TOPORS*  *TOR1AIP1*  *TPK1*  *TPP1*  *TRAF7*  *TRAPPC6A*  *TREX1*  *TRIM32*  *TRIT1*  *TRMT10C*  *TRMT5*  *TRMU*  *TRNT1*  *TRPM1*  *TSFM*  *TSPAN12*  *TTC14*  *TTC19*  *TTC21B*  *TTC8*  *TTLL5*  *TTPA*  *TTR*  *TUB*  *TUBB4B*  *TUBGCP4*  *TUBGCP6*  *TUFM*  *TULP1*  *TWNK*  *TYMP*  *TYR*  *TYRP1*  *TXNRD2*  *UBIAD1*  *UCHL1*  *UNC119*  *UNC13A*  *UNC45B*  *UPF3B*  *UQCC1*  *UQCC2*  *UQCC3*  *UQCR10*  *UQCR11*  *UQCRB*  *UQCRC2*  *UQCRQ*  *USH1C*  *USH1G*  *USH2A*  *VAMP1*  *VARS2*  *VAX1*  *VAX2*  *VCAN*  *VHL*  *VIM*  *VPS13B*  *VSX1*  *VSX2*  *WARS2*  *WDPCP*  *WDR17*  *WDR19*  *DYNC2I2*  *WDR35*  *WDR36*  *WDR73*  *WDR87*  *WFS1*  *WHRN*  *WRN*  *XPNPEP3*  *XYLT2*  *YARS2*  *YBX1*  *YME1L1*  *ZEB2*  *ZFYVE26*  *ZIC3*  *ZNF408*  *ZNF423*  *ZNF469*  *ZNF513*  *ZNHIT3* | *ABCA4*  *ABCC6*  *ABHD12*  *ACBD5*  *ACO2*  *ADAM9*  *ADAMTS18*  *ADGRA3*  *ADGRV1*  *ADIPOR1*  *AFG3L2*  *AGBL5*  *AHI1*  *AHR*  *AIPL1*  *ALMS1*  *ARHGEF18*  *ARL2BP*  *ARL3*  *ARL6*  *ARMS2*  *ARSG*  *ASRGL1*  *ATF6*  *ATOH7*  *ATXN7*  *BBIP1*  *BBS1*  *BBS10*  *BBS12*  *BBS2*  *BBS4*  *BBS5*  *BBS7*  *BBS9*  *BEST1*  *C1QTNF5*  *C2*  *C3*  *CA4*  *CABP4*  *CACNA1F*  *CACNA2D4*  *CAPN5*  *CC2D2A*  *CCDC51*  *CCT2*  *CDH23*  *CDH3*  *CDHR1*  *CEP162*  *CEP164*  *CEP19*  *CEP250*  *CEP290*  *CEP78*  *CERKL*  *CFAP410*  *CFAP418*  *CFB*  *CFH*  *CHM*  *CIB2*  *CISD2*  *CLCC1*  *CLCN2*  *CLEC3B*  *CLN3*  *CLRN1*  *CLUAP1*  *CNGA1*  *CNGA3*  *CNGB1*  *CNGB3*  *CNNM4*  *COL11A1*  *COL2A1*  *COL9A1*  *COQ2*  *COQ4*  *COQ5*  *COQ8B*  *CRB1*  *CRX*  *CSPP1*  *CTNNA1*  *CWC27*  *CYP4V2*  *DHDDS*  *DHX38*  *DMD*  *DNM1L*  *DRAM2*  *DTHD1*  *DYNC2H1*  *DYNC2I2*  *EFEMP1*  *ELOVL1*  *ELOVL4*  *EMC1*  *ENSA*  *ERCC6*  *ESPN*  *EXOSC2*  *EYS*  *FAM161A*  *FBLN5*  *FLVCR1*  *FSCN2*  *FZD4*  *GDF6*  *GNAT1*  *GNAT2*  *GNB3*  *GNPTG*  *GPR179*  *GRK1*  *GRM6*  *GUCA1A*  *GUCA1B*  *GUCY2D*  *HARS1*  *HGSNAT*  *HK1*  *HKDC1*  *HMCN1*  *HMX1*  *HTRA1*  *IDH3B*  *IFT140*  *IFT172*  *IFT27*  *IFT43*  *IFT81*  *IMPDH1*  *IMPG1*  *IMPG2*  *INPP5E*  *INVS*  *IQCB1*  *ITM2B*  *JAG1*  *KCNJ13*  *KCNV2*  *KIAA1549*  *KIF11*  *KIF3B*  *KIZ*  *KLHL7*  *LAMA1*  *LCA5*  *LRAT*  *LRIT3*  *LRP5*  *LRRTM4*  *LZTFL1*  *MAK*  *MAPKAPK3*  *MERTK*  *MFN2*  *MFRP*  *MFSD8*  *MIEF1*  *MIR204*  *MKKS*  *MKS1*  *MMP19*  *MPDZ*  *MTRFR*  *MTTP*  *MVK*  *MYO7A*  *NBAS*  *NDP*  *NEK2*  *NEUROD1*  *NMNAT1*  *NPHP1*  *NPHP3*  *NPHP4*  *NR2E3*  *NR2F1*  *NRL*  *NYX*  *OAT*  *OFD1*  *OPA1*  *OPA3*  *OPN1LW*  *OPN1MW*  *OPN1SW*  *OR2W3*  *OTX2*  *PANK2*  *PAX2*  *PCARE*  *PCDH15*  *PCYT1A*  *PDE6A*  *PDE6B*  *PDE6C*  *PDE6G*  *PDE6H*  *PDSS1*  *PDZD7*  *PEX1*  *PEX2*  *PEX7*  *PGK1*  *PHYH*  *PITPNM3*  *PLA2G5*  *PLK4*  *PNPLA6*  *POC1B*  *POC5*  *POMGNT1*  *PPT1*  *PRCD*  *PRDM13*  *PROM1*  *PROS1*  *PRPF3*  *PRPF31*  *PRPF4*  *PRPF6*  *PRPF8*  *PRPH2*  *PRPS1*  *RAB28*  *RAX2*  *RB1*  *RBP3*  *RBP4*  *RCBTB1*  *RD3*  *RDH11*  *RDH12*  *RDH5*  *REEP6*  *RGR*  *RGS9*  *RGS9BP*  *RHO*  *RIMS1*  *RIMS2*  *RLBP1*  *ROM1*  *RP1*  *RP1L1*  *RP2*  *RP9*  *RPE65*  *RPGR*  *RPGRIP1*  *RPGRIP1L*  *RS1*  *RTN4IP1*  *SAG*  *SAMD11*  *SAMD7*  *SDCCAG8*  *SEMA4A*  *SLC24A1*  *SLC25A46*  *SLC37A3*  *SLC38A8*  *SLC39A12*  *SLC4A7*  *SLC66A1*  *SLC7A14*  *SNRNP200*  *SPATA7*  *SPP2*  *SUMF1*  *TBC1D32*  *TEAD1*  *TIMM8A*  *TIMP3*  *TLCD3B*  *TLR3*  *TLR4*  *TMEM126A*  *TMEM216*  *TMEM237*  *TOPORS*  *TREX1*  *TRIM32*  *TRNT1*  *TRPM1*  *TSPAN12*  *TTC8*  *TTLL5*  *TTPA*  *TUB*  *TUBGCP4*  *TUBGCP6*  *TULP1*  *UBAP1L*  *UNC119*  *USH1C*  *USH1G*  *USH2A*  *USP45*  *VCAN*  *VWA8*  *WDPCP*  *WDR19*  *WFS1*  *WHRN*  *ZNF408*  *ZNF423*  *ZNF513* | *ABCA4*  *ABCC6*  *ABHD12*  *ACBD5*  *ACO2*  *ADAM9*  *ADAMTS18*  *ADGRV1*  *AFG3L2*  *AGBL5*  *AHI1*  *AIPL1*  *AIRE*  *ALDH3A2*  *ALMS1*  *ALPK1*  *AMACR*  *ARHGEF18*  *ARL13B*  *ARL2BP*  *ARL3*  *ARL6*  *ARSG*  *ATF6*  *ATOH7*  *ATXN7*  *BBS1*  *BBS10*  *BBS12*  *BBS2*  *BBS4*  *BBS5*  *BBS7*  *BBS9*  *BEST1*  *C1QTNF5*  *CABP4*  *CACNA1F*  *CACNA2D4*  *CAPN5*  *CC2D2A*  *CDH23*  *CDH3*  *CDHR1*  *CEP164*  *CEP250*  *CEP290*  *CEP78*  *CERKL*  *CFAP20*  *CFAP410*  *CFAP418*  *CFH*  *CHM*  *CLN3*  *CLN5*  *CLN6*  *CLN8*  *CLRN1*  *CNGA1*  *CNGA3*  *CNGB1*  *CNGB3*  *CNNM4*  *COL11A1*  *COL18A1*  *COL2A1*  *COL4A1*  *COL9A1*  *COL9A2*  *COL9A3*  *COQ2*  *CRB1*  *CRX*  *CSPP1*  *CTC1*  *CTNNA1*  *CTNNB1*  *CTNND1*  *CTSD*  *CWC27*  *CYP4V2*  *DHDDS*  *DRAM2*  *DYNC2H1*  *EFEMP1*  *ELOVL4*  *ERCC6*  *ERCC8*  *EYS*  *FAM161A*  *FLVCR1*  *FZD4*  *GNAT1*  *GNAT2*  *GNB3*  *GNPTG*  *GPR143*  *GPR179*  *GRK1*  *GRM6*  *GRN*  *GUCA1A*  *GUCA1B*  *GUCY2D*  *HCCS*  *HGSNAT*  *HK1*  *HMX1*  *IDH3A*  *IDH3B*  *IFT140*  *IFT172*  *IFT27*  *IFT74*  *IKBKG*  *IMPDH1*  *IMPG1*  *IMPG2*  *INPP5E*  *IQCB1*  *JAG1*  *KCNJ13*  *KCNV2*  *KIAA1549*  *KIF11*  *KIZ*  *KLHL7*  *LAMA1*  *LAMP2*  *LCA5*  *LRAT*  *LRIT3*  *LRP2*  *LRP5*  *LZTFL1*  *MAK*  *MCOLN1*  *MED12*  *MERTK*  *MFRP*  *MFSD8*  *MIR204*  *MKKS*  *MKS1*  *MMACHC*  *MPDZ*  *MSTO1*  *MTTP*  *MVK*  *MYO7A*  *NBAS*  *NDP*  *NEUROD1*  *NMNAT1*  *NPHP1*  *NPHP3*  *NPHP4*  *NR2E3*  *NRL*  *NYX*  *OAT*  *OFD1*  *OPN1LW*  *OPN1MW*  *OTX2*  *P3H2*  *PANK2*  *PAX2*  *PCARE*  *PCDH15*  *PCYT1A*  *PDE6A*  *PDE6B*  *PDE6C*  *PDE6G*  *PDSS1*  *PEX1*  *PEX2*  *PEX6*  *PEX7*  *PHYH*  *PLA2G5*  *PLK4*  *PNPLA6*  *POC1B*  *POMGNT1*  *POMT1*  *PPT1*  *PRCD*  *PRDM13*  *PROM1*  *PRPF3*  *PRPF31*  *PRPF4*  *PRPF6*  *PRPF8*  *PRPH2*  *PRPS1*  *PYGM*  *RAB28*  *RAX2*  *RBP3*  *RBP4*  *RCBTB1*  *RD3*  *RDH12*  *RDH5*  *REEP6*  *RGR*  *RGS9*  *RHO*  *RIMS2*  *RLBP1*  *RNU4ATAC*  *ROM1*  *RP1*  *RP1L1*  *RP2*  *RP9*  *RPE65*  *RPGR*  *RPGRIP1*  *RPGRIP1L*  *RS1*  *SAG*  *SAMD7*  *SCAPER*  *SDCCAG8*  *SGSH*  *SLC24A1*  *SLC37A3*  *SLC38A8*  *SLC66A1*  *SLC6A6*  *SNRNP200*  *SPATA7*  *SRD5A3*  *SSBP1*  *STN1*  *SUMF1*  *TIMM8A*  *TIMP3*  *TINF2*  *TLCD3B*  *TMEM216*  *TMEM218*  *TMEM231*  *TMEM237*  *TOPORS*  *TPP1*  *TRAF3IP1*  *TREX1*  *TRNT1*  *TRPM1*  *TSPAN12*  *TTC21B*  *TTC8*  *TTLL5*  *TUB*  *TUBB4B*  *TUBGCP4*  *TUBGCP6*  *TULP1*  *UBAP1L*  *UNC119*  *USH1C*  *USH1G*  *USH2A*  *USP45*  *VCAN*  *VPS13B*  *WDPCP*  *WDR19*  *WHRN*  *ZFYVE26*  *ZNF408*  *ZNF423* | *AARS2*  *AASS*  *ABAT*  *ABCA1*  *ABCA3*  *ABCB6*  *ABCB7*  *ABCD1*  *ACACA*  *ACACB*  *ACAD9*  *ACADL*  *ACADM*  *ACADS*  *ACADVL*  *ACAT1*  *ADAMTS10*  *ADAMTS17*  *ADAMTS2*  *ADAMTSL4*  *AFAP1*  *AFG2A*  *AGK*  *AGPAT1*  *AGPS*  *AGRN*  *AIFM1*  *AKAP14*  *AKR1C1*  *ALAS2*  *ALDH18A1*  *ALDH1A3*  *ALG1*  *ALG14*  *ALG2*  *ALPL*  *ANGPT1*  *ANKS6*  *ANO10*  *ANTXR1*  *AP3B1*  *AP3D1*  *AP5Z1*  *APC*  *APOB*  *APTX*  *ARFGAP2*  *ARHGEF12*  *ARID1A*  *ARL2*  *ARMC9*  *ASB10*  *ASIC5*  *ASPH*  *ATAD3A*  *ATOH1*  *ATP13A2*  *ATP1A3*  *ATP5F1A*  *ATP5F1D*  *ATP5F1E*  *ATP5MF*  *ATP5MG*  *ATP5MGL*  *ATP5PB*  *ATP5PD*  *ATP7B*  *ATPAF1*  *ATPAF2*  *ATXN2*  *AUH*  *B3GALNT2*  *B3GALT6*  *B3GLCT*  *B4GALT7*  *B4GAT1*  *B9D1*  *B9D2*  *BCKDHA*  *BCKDHB*  *BCO1*  *BCO2*  *BCOR*  *BCS1L*  *BFSP1*  *BFSP2*  *BLOC1S3*  *BLOC1S5*  *BLOC1S6*  *BMP4*  *BMP7*  *BOLA3*  *BTD*  *BUB1B*  *C12ORF57*  *C19ORF12*  *C1QBP*  *C1R*  *C2CD3*  *C8A*  *C8B*  *C9*  *CA5A*  *CACNA1A*  *CANT1*  *CAPN15*  *CARS2*  *CASK*  *CAV1*  *CAV2*  *CBS*  *CCDC28B*  *CCDC39*  *CCDC40*  *CCER1*  *CCND1*  *CCNP*  *CD36*  *CDK5RAP2*  *CDKN2A*  *CDKN2B-AS1*  *CEP104*  *CEP120*  *CEP41*  *CEP83*  *CFHR1*  *CFHR2*  *CFHR3*  *CFI*  *CHAT*  *CHCHD10*  *CHD7*  *CHD8*  *CHERP*  *CHKB*  *CHMP4B*  *CHN1*  *CHRNA1*  *CHRNB1*  *CHRND*  *CHRNE*  *CHST14*  *CHST6*  *CLDN19*  *CLPB*  *CLPP*  *CLTA*  *CNTNAP2*  *COA3*  *COA4*  *COA5*  *COA6*  *COA7*  *COA8*  *COG4*  *COG6*  *COL11A2*  *COL12A1*  *COL13A1*  *COL1A1*  *COL1A2*  *COL26A1*  *COL3A1*  *COL4A2*  *COL4A3*  *COL4A4*  *COL4A5*  *COL5A1*  *COL5A2*  *COL8A1*  *COL8A2*  *COLQ*  *COQ6*  *COQ7*  *COQ8A*  *COQ9*  *COX10*  *COX11*  *COX14*  *COX15*  *COX16*  *COX17*  *COX18*  *COX19*  *COX20*  *COX4I1*  *COX4I2*  *COX6A1*  *COX6A2*  *COX6B1*  *COX6B2*  *COX7A1*  *COX7B*  *COX8A*  *CPAMD8*  *CPLANE1*  *CPT1A*  *CPT2*  *CREBBP*  *CRELD1*  *CRPPA*  *CRYAA*  *CRYAB*  *CRYBA1*  *CRYBA2*  *CRYBA4*  *CRYBB1*  *CRYBB2*  *CRYBB3*  *CRYGA*  *CRYGB*  *CRYGC*  *CRYGD*  *CRYGS*  *CSMD1*  *CSMD2*  *CST3*  *CTDP1*  *CTSF*  *CX3CR1*  *CYC1*  *CYCS*  *CYP1B1*  *CYP27A1*  *CYP51A1*  *DAG1*  *DARS2*  *DBT*  *DCC*  *DCDC1*  *DCN*  *DCT*  *DDHD2*  *DGUOK*  *DHCR7*  *DHX32*  *DLAT*  *DLD*  *DNA2*  *DNAAF1*  *DNAAF11*  *DNAAF19*  *DNAAF2*  *DNAAF3*  *DNAAF5*  *DNAH11*  *DNAH5*  *DNAI1*  *DNAI2*  *DNAJC17*  *DNAJC19*  *DNAJC30*  *DNAJC5*  *DNAL1*  *DNM2*  *DOCK6*  *DOK7*  *DPAGT1*  *DSCAML1*  *DSE*  *DTNBP1*  *EARS2*  *ECHS1*  *EDN3*  *EDNRB*  *ELAC2*  *ELP1*  *ELP4*  *EPG5*  *EPHA2*  *ERAL1*  *ERCC1*  *ERCC2*  *ERCC5*  *ESCO2*  *ETFA*  *ETFB*  *ETFDH*  *ETHE1*  *EVA1A*  *EVC*  *EVC2*  *EXO5*  *EYA1*  *FA2H*  *FAM131A*  *FARS2*  *FASTKD2*  *FBN1*  *FBN2*  *FBP1*  *FBXL4*  *FCN1*  *FDFT1*  *FDX2*  *FDXR*  *FERMT1*  *FGF21*  *FH*  *FKBP14*  *FKRP*  *FKTN*  *FLAD1*  *FLNB*  *FOXC1*  *FOXC2*  *FOXE3*  *FOXH1*  *FOXRED1*  *FRAS1*  *FREM2*  *FRMD7*  *FTL*  *FXN*  *FYCO1*  *FZD5*  *G6PC1*  *GAA*  *GALE*  *GALK1*  *GALM*  *GALT*  *GAMT*  *GAN*  *GARS1*  *GAS7*  *GATB*  *GATC*  *GATM*  *GBA2*  *GCDH*  *GCNT2*  *GDAP1*  *GDF1*  *GDF3*  *GFER*  *GFM1*  *GFM2*  *GFPT1*  *GJA1*  *GJA3*  *GJA8*  *GJB1*  *GJB2*  *GJB6*  *GJC3*  *GLA*  *GLIS2*  *GLRX5*  *GMDS*  *GMPPB*  *GNPAT*  *GNS*  *GPR160*  *GPR45*  *GRIP1*  *GRWD1*  *GTPBP3*  *GUCA1C*  *GYS2*  *GZF1*  *HADH*  *HADHA*  *HADHB*  *HARS2*  *HCN1*  *HESX1*  *HGF*  *HIBCH*  *HLCS*  *HMGB3*  *HMGCL*  *HMGCS2*  *HPS1*  *HPS3*  *HPS4*  *HPS5*  *HPS6*  *HSD17B10*  *HSF4*  *HSPD1*  *HTRA2*  *HYCC1*  *HYLS1*  *IARS1*  *IARS2*  *IBA57*  *IDUA*  *IFT122*  *IFT52*  *IFT80*  *IFT88*  *INPP5B*  *INTS1*  *INTS15*  *ISCA1*  *ISCA2*  *ISCU*  *JAM3*  *KARS1*  *KATNIP*  *KCTD7*  *KERA*  *KIAA0586*  *KIF1A*  *KIF24*  *KIF7*  *KIT*  *KLC2*  *KLHL21*  *LAMA5*  *LAMB1*  *LAMB2*  *LARGE1*  *LARS1*  *LARS2*  *LCT*  *LDLR*  *LEFTY2*  *LEMD2*  *LENG8*  *LHX2*  *LIAS*  *LIM2*  *LIPT1*  *LIPT2*  *LMX1B*  *LONP1*  *LOXL1*  *LOXL3*  *LRMDA*  *LRP4*  *LRPPRC*  *LRRC32*  *LSS*  *LTBP2*  *LTBP3*  *LYRM4*  *LYRM7*  *LYST*  *MAB21L1*  *MAB21L2*  *MACF1*  *MAF*  *MAN1C1*  *MAN2B1*  *MAP6D1*  *MAPKBP1*  *MARS2*  *MASP1*  *MASP2*  *MC1R*  *MCAT*  *MDH2*  *MECR*  *MEIS1*  *MFF*  *MGME1*  *MICOS13*  *MICU1*  *MIP*  *MIPEP*  *MIR184*  *MITF*  *MLPH*  *MMP1*  *MMP9*  *MORC2*  *MPC1*  *MPRIP*  *MPV17*  *MRM2*  *MRPL12*  *MRPL3*  *MRPL44*  *MRPS14*  *MRPS16*  *MRPS2*  *MRPS22*  *MRPS23*  *MRPS34*  *MRPS7*  *MRRF*  *MRS2*  *MSRA*  *MTFMT*  *MTMR7*  *MTO1*  *MTPAP*  *MUSK*  *MXRA8*  *MYH9*  *MYO5A*  *MYO9A*  *MYOC*  *NAA10*  *NADK2*  *NARS2*  *NAXE*  *NDRG4*  *NDUFA1*  *NDUFA10*  *NDUFA11*  *NDUFA12*  *NDUFA13*  *NDUFA2*  *NDUFA4*  *NDUFA6*  *NDUFA7*  *NDUFA8*  *NDUFA9*  *NDUFAF1*  *NDUFAF2*  *NDUFAF3*  *NDUFAF4*  *NDUFAF5*  *NDUFAF6*  *NDUFAF7*  *NDUFAF8*  *NDUFB11*  *NDUFB3*  *NDUFB6*  *NDUFB8*  *NDUFB9*  *NDUFS1*  *NDUFS2*  *NDUFS3*  *NDUFS4*  *NDUFS5*  *NDUFS6*  *NDUFS7*  *NDUFS8*  *NDUFV1*  *NDUFV2*  *NDUFV3*  *NECTIN2*  *NECTIN3*  *NEFH*  *NEK1*  *NEK8*  *NF2*  *NFS1*  *NFU1*  *NHS*  *NIPAL3*  *NKX2-5*  *NME8*  *NOD2*  *NODAL*  *NOS3*  *NOTCH2*  *NOTCH3*  *NRCAM*  *NSUN3*  *NT5C2*  *NTF4*  *NUBPL*  *NUTF2*  *NXNL1*  *OCA2*  *OCRL*  *ODAD1*  *OPTC*  *OPTN*  *OTC*  *OTOGL*  *OXA1L*  *PARS2*  *PAX3*  *PAX6*  *PC*  *PCCA*  *PCCB*  *PCK2*  *PCLO*  *PDE6D*  *PDHA1*  *PDHB*  *PDHX*  *PDP1*  *PDSS2*  *PDX1*  *PDXK*  *PEAK1*  *PET100*  *PET117*  *PEX10*  *PEX11B*  *PEX12*  *PEX13*  *PEX14*  *PEX16*  *PEX19*  *PEX26*  *PEX3*  *PEX5*  *PIBF1*  *PIK3R1*  *PITRM1*  *PITX2*  *PITX3*  *PKM*  *PLA2G6*  *PLD1*  *PLD3*  *PLEC*  *PLOD1*  *PLOD3*  *PLP1*  *PMP22*  *PMPCA*  *PMPCB*  *PNPLA2*  *PNPLA8*  *PNPT1*  *POLG*  *POLG2*  *POLRMT*  *POMGNT2*  *POMK*  *POMP*  *POMT2*  *PON2*  *PORCN*  *PPA2*  *PRDM5*  *PREPL*  *PRICKLE3*  *PRKCI*  *PROX1*  *PRR12*  *PRSS56*  *PRX*  *PTCD1*  *PTCD3*  *PTCH1*  *PTPRU*  *PUS1*  *PXDN*  *QARS1*  *QRSL1*  *RAB18*  *RAB27A*  *RAB3GAP1*  *RAB3GAP2*  *RAPSN*  *RARB*  *RARS1*  *RARS2*  *RAX*  *RBMS3*  *RECQL4*  *REEP1*  *RERE*  *RGS6*  *RHEX*  *RHOBTB2*  *RIGI*  *RMND1*  *RNASEH1*  *RNLS*  *RPH3A*  *RPL10*  *RPL15*  *RPS19*  *RRAGA*  *RRM2B*  *RSPH4A*  *RSPH9*  *RTBDN*  *RYR1*  *SACS*  *SALL2*  *SARS2*  *SBF2*  *SC5D*  *SCARF2*  *SCLT1*  *SCN4A*  *SCO1*  *SCO2*  *SDHA*  *SDHAF1*  *SDHAF2*  *SDHAF3*  *SDHAF4*  *SDHB*  *SDHC*  *SDHD*  *SEC23A*  *SEMA3A*  *SEMA6A*  *SERAC1*  *SETX*  *SFXN4*  *SH3PXD2B*  *SHH*  *SIL1*  *SIPA1L3*  *SIX1*  *SIX3*  *SIX5*  *SIX6*  *SLC16A12*  *SLC18A3*  *SLC19A2*  *SLC19A3*  *SLC1A5*  *SLC22A5*  *SLC24A5*  *SLC25A1*  *SLC25A12*  *SLC25A13*  *SLC25A15*  *SLC25A19*  *SLC25A20*  *SLC25A21*  *SLC25A26*  *SLC25A3*  *SLC25A32*  *SLC25A33*  *SLC25A38*  *SLC25A4*  *SLC25A40*  *SLC25A42*  *SLC2A1*  *SLC33A1*  *SLC37A4*  *SLC39A13*  *SLC44A1*  *SLC45A2*  *SLC4A4*  *SLC52A2*  *SLC5A7*  *SLC6A15*  *SLC6A8*  *SMOC1*  *SNAI2*  *SNAP25*  *SNX10*  *SNX3*  *SOD1*  *SOX1*  *SOX10*  *SOX2*  *SOX3*  *SPAST*  *SPG11*  *SPG7*  *SPINT2*  *SREBF2*  *STARD9*  *STAT2*  *STEAP1*  *STEAP2*  *STK38L*  *STRA6*  *SUCLA2*  *SUCLG1*  *SUFU*  *SUOX*  *SURF1*  *SYNE2*  *SYT2*  *SYTL4*  *TACO1*  *TACR1*  *TARS2*  *TBC1D20*  *TBCE*  *TBK1*  *TCTN1*  *TCTN2*  *TCTN3*  *TDRD7*  *TEK*  *TENM3*  *TFAM*  *TFAP2A*  *TFB1M*  *TFG*  *TGFBI*  *THBS1*  *TIMM22*  *TIMM50*  *TIMMDC1*  *TK2*  *TKFC*  *TLCD5*  *TMCO1*  *TMED3*  *TMED7*  *TMEM107*  *TMEM114*  *TMEM126B*  *TMEM138*  *TMEM65*  *TMEM67*  *TMEM70*  *TMEM98*  *TNPO1*  *TNXB*  *TOP3A*  *TOR1AIP1*  *TPK1*  *TRAF7*  *TRAPPC6A*  *TRIT1*  *TRMT10C*  *TRMT5*  *TRMU*  *TSFM*  *TTC14*  *TTC19*  *TTR*  *TUFM*  *TWNK*  *TXNRD2*  *TYMP*  *TYR*  *TYRP1*  *UBIAD1*  *UCHL1*  *UNC13A*  *UNC45B*  *UPF3B*  *UQCC1*  *UQCC2*  *UQCC3*  *UQCR10*  *UQCR11*  *UQCRB*  *UQCRC2*  *UQCRQ*  *VAMP1*  *VARS2*  *VAX1*  *VAX2*  *VHL*  *VIM*  *VSX1*  *VSX2*  *WARS2*  *WDR17*  *WDR35*  *WDR36*  *WDR73*  *WDR87*  *WRN*  *WWTR1*  *XPNPEP3*  *XYLT2*  *YARS2*  *YBX1*  *YME1L1*  *ZEB2*  *ZIC3*  *ZNF469*  *ZNHIT3* | *CCDC51*  *CEP162*  *CLCN2*  *LRRTM4*  *MMP19*  *SLC39A12*  *VWA8* | *AIRE*  *CFAP20*  *CTC1*  *IKBKG*  *MCOLN1*  *MED12*  *RNU4ATAC*  *SGSH*  *SLC6A6*  *STN1*  *TINF2* | *ADGRA3*  *ADIPOR1*  *AHR*  *ARMS2*  *ASRGL1*  *BBIP1*  *C2*  *C3*  *CA4*  *CCT2*  *CEP19*  *CFB*  *CIB2*  *CISD2*  *CLCC1*  *CLEC3B*  *CLUAP1*  *COQ4*  *COQ5*  *COQ8B*  *DHX38*  *DMD*  *DNM1L*  *DTHD1*  *DYNC2I2*  *ELOVL1*  *EMC1*  *ENSA*  *ESPN*  *EXOSC2*  *FBLN5*  *FSCN2*  *GDF6*  *HARS1*  *HKDC1*  *HMCN1*  *HTRA1*  *IFT43*  *IFT81*  *INVS*  *ITM2B*  *KIF3B*  *MAPKAPK3*  *MFN2*  *MIEF1*  *MTRFR*  *NEK2*  *NR2F1*  *OPA1*  *OPA3*  *OPN1SW*  *OR2W3*  *PDE6H*  *PDZD7*  *PGK1*  *PITPNM3*  *POC5*  *PROS1*  *RB1*  *RDH11*  *RGS9BP*  *RIMS1*  *RTN4IP1*  *SAMD11*  *SEMA4A*  *SLC25A46*  *SLC4A7*  *SLC7A14*  *SPP2*  *TBC1D32*  *TEAD1*  *TLR3*  *TLR4*  *TMEM126A*  *TRIM32*  *TTPA*  *WFS1*  *ZNF513* | *ALDH3A2*  *ALPK1*  *AMACR*  *ARL13B*  *CLN5*  *CLN6*  *CLN8*  *COL18A1*  *COL4A1*  *COL9A2*  *COL9A3*  *CTNNB1*  *CTNND1*  *CTSD*  *ERCC8*  *GPR143*  *GRN*  *HCCS*  *IDH3A*  *IFT74*  *LAMP2*  *LRP2*  *MMACHC*  *MSTO1*  *P3H2*  *PEX6*  *POMT1*  *PYGM*  *SCAPER*  *SRD5A3*  *SSBP1*  *TMEM218*  *TMEM231*  *TPP1*  *TRAF3IP1*  *TTC21B*  *TUBB4B*  *VPS13B*  *ZFYVE26* | *MPDZ*  *SLC66A*  *SAMD7*  *SLC37A3*  *SUMF1*  *UBAP1L*  *USP45* | *ABCA4*  *ABCC6*  *ABHD12*  *ACBD5*  *ACO2*  *ADAM9*  *ADAMTS18*  *ADGRV1*  *AFG3L2*  *AGBL5*  *AHI1*  *AIPL1*  *ALMS1*  *ARHGEF18*  *ARL2BP*  *ARL3*  *ARL6*  *ARSG*  *ATF6*  *ATOH7*  *ATXN7*  *BBS1*  *BBS10*  *BBS12*  *BBS2*  *BBS4*  *BBS5*  *BBS7*  *BBS9*  *BEST1*  *C1QTNF5*  *CABP4*  *CACNA1F*  *CACNA2D4*  *CAPN5*  *CC2D2A*  *CDH23*  *CDH3*  *CDHR1*  *CEP164*  *CEP250*  *CEP290*  *CEP78*  *CERKL*  *CFAP410*  *CFAP418*  *CFH*  *CHM*  *CLN3*  *CLRN1*  *CNGA1*  *CNGA3*  *CNGB1*  *CNGB3*  *CNNM4*  *COL11A1*  *COL2A1*  *COL9A1*  *COQ2*  *CRB1*  *CRX*  *CSPP1*  *CTNNA1*  *CWC27*  *CYP4V2*  *DHDDS*  *DRAM2*  *DYNC2H1*  *EFEMP1*  *ELOVL4*  *ERCC6*  *EYS*  *FAM161A*  *FLVCR1*  *FZD4*  *GNAT1*  *GNAT2*  *GNB3*  *GNPTG*  *GPR179*  *GRK1*  *GRM6*  *GUCA1A*  *GUCA1B*  *GUCY2D*  *HGSNAT*  *HMX1*  *IDH3B*  *IFT140*  *IFT172*  *IFT27*  *IMPDH1*  *IMPG1*  *IMPG2*  *INPP5E*  *IQCB1*  *JAG1*  *KCNJ13*  *KCNV2*  *KIAA1549*  *KIF11*  *KIZ*  *KLHL7*  *LAMA1*  *LCA5*  *LRAT*  *LRIT3*  *LRP5*  *LZTFL1*  *MAK*  *MERTK*  *MFRP*  *MFSD8*  *MIR204*  *MKKS*  *MKS1*  *MTTP*  *MVK*  *MYO7A*  *NBAS*  *NDP*  *NEUROD1*  *NMNAT1*  *NPHP1*  *NPHP3*  *NPHP4*  *NR2E3*  *NRL*  *NYX*  *OAT*  *OFD1*  *OPN1LW*  *OPN1MW*  *OTX2*  *PANK2*  *PAX2*  *PCARE*  *PCDH15*  *PCYT1A*  *PDE6A*  *PDE6B*  *PDE6C*  *PDE6G*  *PDSS1*  *PEX1*  *PEX2*  *PEX7*  *PHYH*  *PLA2G5*  *PLK4*  *PNPLA6*  *POC1B*  *POMGNT1*  *PPT1*  *PRCD*  *PRDM13*  *PROM1*  *PRPF3*  *PRPF31*  *PRPF4*  *PRPF6*  *PRPF8*  *PRPH2*  *PRPS1*  *RAB28*  *RAX2*  *RBP3*  *RBP4*  *RCBTB1*  *RD3*  *RDH12*  *RDH5*  *REEP6*  *RGR*  *RGS9*  *RHO*  *RIMS2*  *RLBP1*  *ROM1*  *RP1*  *RP1L1*  *RP2*  *RP9*  *RPE65*  *RPGR*  *RPGRIP1*  *RPGRIP1L*  *RS1*  *SAG*  *SCAPER*  *SDCCAG8*  *SLC24A1*  *SLC38A8*  *SNRNP200*  *SPATA7*  *TIMM8A*  *TIMP3*  *TLCD3B*  *TMEM216*  *TMEM237*  *TOPORS*  *TREX1*  *TRNT1*  *TRPM1*  *TSPAN12*  *TTC8*  *TTLL5*  *TUB*  *TUBGCP4*  *TUBGCP6*  *TULP1*  *UNC119*  *USH1C*  *USH1G*  *USH2A*  *VCAN*  *WDPCP*  *WDR19*  *WHRN*  *ZNF408*  *ZNF423* |
