# Supplementary material for: Comparison of inherited retinal disease genes covered by two comprehensive genetic testing panels and a widely used online resource
Source: Eye (Lond). 2025 Jan 28;39(5):1009–10. doi: 10.1038/s41433-025-03629-0 (PMC11933294; doi:10.1038/s41433-025-03629-0)
Supplement: Supplementary file 2 — Supplemental Table 2 [file 41433_2025_3629_MOESM2_ESM.docx]

**Supplementary Table 2. Genes in each list, unique to each list, or overlapping between lists, when only including MVL-designated retinal dystrophy genes (for MVL).** Genes in each list are following curation (exclusion of duplicate or alternative non-approved gene symbols, exclusion of mitochondrial genes, exclusion of loci with gene not yet identified). MVL (Ret Dys) refers to Molecular Vision Laboratory (MVL) “Vision Panel”, Version 21.2 (<https://www.molecularvisionlab.com/mvl-vision-panel/> accessed 8 Dec 2024), but only listing those genes included in a list on the MVL website as being associated with retinal dystrophy or IRD (although the full panel interrogates >1000 genes). PanelApp refers to approved (“green”) genes for retinal disorders in the Genomics England PanelApp Version 7.0 (<https://panelapp.genomicsengland.co.uk/panels/307/> accessed 8 Dec 2024). RetNet refers to the genes listed in the online “Retinal Information Network” resource (<https://retnet.org/> accessed 8 Dec 2024).

| **Total for each curated list** | | | **Genes unique to each list** | | | **Genes common to 2 lists only** | | | **Genes common to all 3 lists**  (n=194) |
| --- | --- | --- | --- | --- | --- | --- | --- | --- | --- |
| MVL (Ret Dys)  (n=278) | RetNet (n=313) | PanelApp (n=278) | MVL (Ret Dys)  (n=30) | RetNet  (n=44) | PanelApp  (n=37) | Genes in both MVL (Ret Dys) and RetNet, but not in PanelApp  (n=41) | Genes in both MVL (Ret Dys) and PanelApp, but not in RetNet  (n=13) | Genes in PanelApp and RetNet, but not in MVL (Ret Dys)  (n=34) |  |
| *ABCA4*  *ABCC6*  *ABCD1*  *ABHD12*  *ACBD5*  *ACO2*  *ADAM9*  *ADAMTS18*  *ADGRA3*  *ADGRV1*  *AHI1*  *AIPL1*  *ALMS1*  *AMACR*  *ARL13B*  *ARL2BP*  *ARL3*  *ARL6*  *ATF6*  *B9D1*  *B9D2*  *BBIP1*  *BBS1*  *BBS10*  *BBS12*  *BBS2*  *BBS4*  *BBS5*  *BBS7*  *BBS9*  *BEST1*  *C1QTNF5*  *C5ORF42*  *CA4*  *CABP4*  *CACNA1F*  *CACNA2D4*  *CC2D2A*  *CDH23*  *CDH3*  *CDHR1*  *CEP164*  *CEP250*  *CEP290*  *CEP41*  *CERKL*  *CFAP410*  *CFAP418*  *CFH*  *CHM*  *CIB2*  *CISD2*  *CKAP4*  *CLN13*  *CLN14*  *CLN3*  *CLN5*  *CLN6*  *CLN8*  *CLRN1*  *CNGA1*  *CNGA3*  *CNGB1*  *CNGB3*  *CNNM4*  *CRB1*  *CRX*  *CSPP1*  *CTNNA1*  *CTSD*  *CYP4V2*  *DGKQ*  *DHDDS*  *DHX38*  *DNAJC5*  *DRAM2*  *DTHD1*  *EFEMP1*  *ELOVL4*  *EMC1*  *EYS*  *FAM161A*  *FLVCR1*  *FSCN2*  *FZD4*  *GDF6*  *GJB2*  *GJB6*  *GNAT1*  *GNAT2*  *GPR179*  *GRK1*  *GRM6*  *GRN*  *GUCA1A*  *GUCA1B*  *GUCY2D*  *HARS1*  *HCN1*  *HGSNAT*  *HK1*  *HMCN1*  *HMX1*  *IDH3B*  *IFT140*  *IFT172*  *IFT27*  *IFT81*  *IMPDH1*  *IMPG1*  *IMPG2*  *INPP5E*  *INVS*  *IQCB1*  *ITM2B*  *JAG1*  *KCNJ13*  *KCNV2*  *KIAA1549*  *KIF11*  *KIF7*  *KIZ*  *KLHL7*  *LAMA1*  *LCA5*  *LRAT*  *LRIT3*  *LRP5*  *LZTFL1*  *MAK*  *MERTK*  *MFN2*  *MFRP*  *MFSD8*  *MIR204*  *MKKS*  *MKS1*  *MMACHC*  *MTRFR*  *MTTP*  *MVK*  *MYO7A*  *NDP*  *NEK2*  *NEUROD1*  *NMNAT1*  *NPHP1*  *NPHP3*  *NPHP4*  *NR2E3*  *NR2F1*  *NRL*  *NYX*  *OAT*  *OFD1*  *OPA1*  *OPA3*  *OPN1LW*  *OPN1MW*  *OR2W3*  *OTX2*  *PANK2*  *PAX2*  *PCARE*  *PCDH15*  *PDE6A*  *PDE6B*  *PDE6C*  *PDE6D*  *PDE6G*  *PDE6H*  *PDZD7*  *PEX1*  *PEX10*  *PEX11B*  *PEX12*  *PEX13*  *PEX14*  *PEX16*  *PEX19*  *PEX2*  *PEX26*  *PEX3*  *PEX5*  *PEX6*  *PEX7*  *PGK1*  *PHYH*  *PITPNM3*  *PLA2G5*  *PLK4*  *PNPLA6*  *POC1B*  *PPT1*  *PRCD*  *PROM1*  *PRPF3*  *PRPF31*  *PRPF4*  *PRPF6*  *PRPF8*  *PRPH2*  *PRPS1*  *RAB28*  *RAX2*  *RBP3*  *RBP4*  *RD3*  *RDH11*  *RDH12*  *RDH5*  *RGR*  *RGS9*  *RGS9BP*  *RHO*  *RIMS1*  *RLBP1*  *ROM1*  *RP1*  *RP1L1*  *RP2*  *RP9*  *RPE65*  *RPGR*  *RPGRIP1*  *RPGRIP1L*  *RS1*  *RTN4IP1*  *SAG*  *SDCCAG8*  *SEMA4A*  *SLC24A1*  *SLC25A46*  *SLC4A7*  *SLC7A14*  *SNRNP200*  *SPATA7*  *SPP2*  *TCTN1*  *TCTN2*  *TCTN3*  *TEAD1*  *TIMM8A*  *TIMP3*  *TMEM126A*  *TMEM138*  *TMEM216*  *TMEM231*  *TMEM237*  *TMEM67*  *TOPORS*  *TPP1*  *TREX1*  *TRIM32*  *TRNT1*  *TRPM1*  *TSPAN12*  *TTC21B*  *TTC8*  *TTLL5*  *TTPA*  *TUB*  *TUBGCP4*  *TUBGCP6*  *TULP1*  *UNC119*  *USH1C*  *USH1G*  *USH2A*  *VCAN*  *VPS13B*  *WDPCP*  *WDR19*  *WFS1*  *WHRN*  *ZNF408*  *ZNF423*  *ZNF513* | *ABCA4*  *ABCC6*  *ABHD12*  *ACBD5*  *ACO2*  *ADAM9*  *ADAMTS18*  *ADGRA3*  *ADGRV1*  *ADIPOR1*  *AFG3L2*  *AGBL5*  *AHI1*  *AHR*  *AIPL1*  *ALMS1*  *ARHGEF18*  *ARL2BP*  *ARL3*  *ARL6*  *ARMS2*  *ARSG*  *ASRGL1*  *ATF6*  *ATOH7*  *ATXN7*  *BBIP1*  *BBS1*  *BBS10*  *BBS12*  *BBS2*  *BBS4*  *BBS5*  *BBS7*  *BBS9*  *BEST1*  *C1QTNF5*  *C2*  *C3*  *CA4*  *CABP4*  *CACNA1F*  *CACNA2D4*  *CAPN5*  *CC2D2A*  *CCDC51*  *CCT2*  *CDH23*  *CDH3*  *CDHR1*  *CEP162*  *CEP164*  *CEP19*  *CEP250*  *CEP290*  *CEP78*  *CERKL*  *CFAP410*  *CFAP418*  *CFB*  *CFH*  *CHM*  *CIB2*  *CISD2*  *CLCC1*  *CLCN2*  *CLEC3B*  *CLN3*  *CLRN1*  *CLUAP1*  *CNGA1*  *CNGA3*  *CNGB1*  *CNGB3*  *CNNM4*  *COL11A1*  *COL2A1*  *COL9A1*  *COQ2*  *COQ4*  *COQ5*  *COQ8B*  *CRB1*  *CRX*  *CSPP1*  *CTNNA1*  *CWC27*  *CYP4V2*  *DHDDS*  *DHX38*  *DMD*  *DNM1L*  *DRAM2*  *DTHD1*  *DYNC2H1*  *DYNC2I2*  *EFEMP1*  *ELOVL1*  *ELOVL4*  *EMC1*  *ENSA*  *ERCC6*  *ESPN*  *EXOSC2*  *EYS*  *FAM161A*  *FBLN5*  *FLVCR1*  *FSCN2*  *FZD4*  *GDF6*  *GNAT1*  *GNAT2*  *GNB3*  *GNPTG*  *GPR179*  *GRK1*  *GRM6*  *GUCA1A*  *GUCA1B*  *GUCY2D*  *HARS1*  *HGSNAT*  *HK1*  *HKDC1*  *HMCN1*  *HMX1*  *HTRA1*  *IDH3B*  *IFT140*  *IFT172*  *IFT27*  *IFT43*  *IFT81*  *IMPDH1*  *IMPG1*  *IMPG2*  *INPP5E*  *INVS*  *IQCB1*  *ITM2B*  *JAG1*  *KCNJ13*  *KCNV2*  *KIAA1549*  *KIF11*  *KIF3B*  *KIZ*  *KLHL7*  *LAMA1*  *LCA5*  *LRAT*  *LRIT3*  *LRP5*  *LRRTM4*  *LZTFL1*  *MAK*  *MAPKAPK3*  *MERTK*  *MFN2*  *MFRP*  *MFSD8*  *MIEF1*  *MIR204*  *MKKS*  *MKS1*  *MMP19*  *MPDZ*  *MTRFR*  *MTTP*  *MVK*  *MYO7A*  *NBAS*  *NDP*  *NEK2*  *NEUROD1*  *NMNAT1*  *NPHP1*  *NPHP3*  *NPHP4*  *NR2E3*  *NR2F1*  *NRL*  *NYX*  *OAT*  *OFD1*  *OPA1*  *OPA3*  *OPN1LW*  *OPN1MW*  *OPN1SW*  *OR2W3*  *OTX2*  *PANK2*  *PAX2*  *PCARE*  *PCDH15*  *PCYT1A*  *PDE6A*  *PDE6B*  *PDE6C*  *PDE6G*  *PDE6H*  *PDSS1*  *PDZD7*  *PEX1*  *PEX2*  *PEX7*  *PGK1*  *PHYH*  *PITPNM3*  *PLA2G5*  *PLK4*  *PNPLA6*  *POC1B*  *POC5*  *POMGNT1*  *PPT1*  *PRCD*  *PRDM13*  *PROM1*  *PROS1*  *PRPF3*  *PRPF31*  *PRPF4*  *PRPF6*  *PRPF8*  *PRPH2*  *PRPS1*  *RAB28*  *RAX2*  *RB1*  *RBP3*  *RBP4*  *RCBTB1*  *RD3*  *RDH11*  *RDH12*  *RDH5*  *REEP6*  *RGR*  *RGS9*  *RGS9BP*  *RHO*  *RIMS1*  *RIMS2*  *RLBP1*  *ROM1*  *RP1*  *RP1L1*  *RP2*  *RP9*  *RPE65*  *RPGR*  *RPGRIP1*  *RPGRIP1L*  *RS1*  *RTN4IP1*  *SAG*  *SAMD11*  *SAMD7*  *SDCCAG8*  *SEMA4A*  *SLC24A1*  *SLC25A46*  *SLC37A3*  *SLC38A8*  *SLC39A12*  *SLC4A7*  *SLC66A1*  *SLC7A14*  *SNRNP200*  *SPATA7*  *SPP2*  *SUMF1*  *TBC1D32*  *TEAD1*  *TIMM8A*  *TIMP3*  *TLCD3B*  *TLR3*  *TLR4*  *TMEM126A*  *TMEM216*  *TMEM237*  *TOPORS*  *TREX1*  *TRIM32*  *TRNT1*  *TRPM1*  *TSPAN12*  *TTC8*  *TTLL5*  *TTPA*  *TUB*  *TUBGCP4*  *TUBGCP6*  *TULP1*  *UBAP1L*  *UNC119*  *USH1C*  *USH1G*  *USH2A*  *USP45*  *VCAN*  *VWA8*  *WDPCP*  *WDR19*  *WFS1*  *WHRN*  *ZNF408*  *ZNF423*  *ZNF513* | *ABCA4*  *ABCC6*  *ABHD12*  *ACBD5*  *ACO2*  *ADAM9*  *ADAMTS18*  *ADGRV1*  *AFG3L2*  *AGBL5*  *AHI1*  *AIPL1*  *AIRE*  *ALDH3A2*  *ALMS1*  *ALPK1*  *AMACR*  *ARHGEF18*  *ARL13B*  *ARL2BP*  *ARL3*  *ARL6*  *ARSG*  *ATF6*  *ATOH7*  *ATXN7*  *BBS1*  *BBS10*  *BBS12*  *BBS2*  *BBS4*  *BBS5*  *BBS7*  *BBS9*  *BEST1*  *C1QTNF5*  *CABP4*  *CACNA1F*  *CACNA2D4*  *CAPN5*  *CC2D2A*  *CDH23*  *CDH3*  *CDHR1*  *CEP164*  *CEP250*  *CEP290*  *CEP78*  *CERKL*  *CFAP20*  *CFAP410*  *CFAP418*  *CFH*  *CHM*  *CLN3*  *CLN5*  *CLN6*  *CLN8*  *CLRN1*  *CNGA1*  *CNGA3*  *CNGB1*  *CNGB3*  *CNNM4*  *COL11A1*  *COL18A1*  *COL2A1*  *COL4A1*  *COL9A1*  *COL9A2*  *COL9A3*  *COQ2*  *CRB1*  *CRX*  *CSPP1*  *CTC1*  *CTNNA1*  *CTNNB1*  *CTNND1*  *CTSD*  *CWC27*  *CYP4V2*  *DHDDS*  *DRAM2*  *DYNC2H1*  *EFEMP1*  *ELOVL4*  *ERCC6*  *ERCC8*  *EYS*  *FAM161A*  *FLVCR1*  *FZD4*  *GNAT1*  *GNAT2*  *GNB3*  *GNPTG*  *GPR143*  *GPR179*  *GRK1*  *GRM6*  *GRN*  *GUCA1A*  *GUCA1B*  *GUCY2D*  *HCCS*  *HGSNAT*  *HK1*  *HMX1*  *IDH3A*  *IDH3B*  *IFT140*  *IFT172*  *IFT27*  *IFT74*  *IKBKG*  *IMPDH1*  *IMPG1*  *IMPG2*  *INPP5E*  *IQCB1*  *JAG1*  *KCNJ13*  *KCNV2*  *KIAA1549*  *KIF11*  *KIZ*  *KLHL7*  *LAMA1*  *LAMP2*  *LCA5*  *LRAT*  *LRIT3*  *LRP2*  *LRP5*  *LZTFL1*  *MAK*  *MCOLN1*  *MED12*  *MERTK*  *MFRP*  *MFSD8*  *MIR204*  *MKKS*  *MKS1*  *MMACHC*  *MPDZ*  *MSTO1*  *MTTP*  *MVK*  *MYO7A*  *NBAS*  *NDP*  *NEUROD1*  *NMNAT1*  *NPHP1*  *NPHP3*  *NPHP4*  *NR2E3*  *NRL*  *NYX*  *OAT*  *OFD1*  *OPN1LW*  *OPN1MW*  *OTX2*  *P3H2*  *PANK2*  *PAX2*  *PCARE*  *PCDH15*  *PCYT1A*  *PDE6A*  *PDE6B*  *PDE6C*  *PDE6G*  *PDSS1*  *PEX1*  *PEX2*  *PEX6*  *PEX7*  *PHYH*  *PLA2G5*  *PLK4*  *PNPLA6*  *POC1B*  *POMGNT1*  *POMT1*  *PPT1*  *PRCD*  *PRDM13*  *PROM1*  *PRPF3*  *PRPF31*  *PRPF4*  *PRPF6*  *PRPF8*  *PRPH2*  *PRPS1*  *PYGM*  *RAB28*  *RAX2*  *RBP3*  *RBP4*  *RCBTB1*  *RD3*  *RDH12*  *RDH5*  *REEP6*  *RGR*  *RGS9*  *RHO*  *RIMS2*  *RLBP1*  *RNU4ATAC*  *ROM1*  *RP1*  *RP1L1*  *RP2*  *RP9*  *RPE65*  *RPGR*  *RPGRIP1*  *RPGRIP1L*  *RS1*  *SAG*  *SAMD7*  *SCAPER*  *SDCCAG8*  *SGSH*  *SLC24A1*  *SLC37A3*  *SLC38A8*  *SLC66A1*  *SLC6A6*  *SNRNP200*  *SPATA7*  *SRD5A3*  *SSBP1*  *STN1*  *SUMF1*  *TIMM8A*  *TIMP3*  *TINF2*  *TLCD3B*  *TMEM216*  *TMEM218*  *TMEM231*  *TMEM237*  *TOPORS*  *TPP1*  *TRAF3IP1*  *TREX1*  *TRNT1*  *TRPM1*  *TSPAN12*  *TTC21B*  *TTC8*  *TTLL5*  *TUB*  *TUBB4B*  *TUBGCP4*  *TUBGCP6*  *TULP1*  *UBAP1L*  *UNC119*  *USH1C*  *USH1G*  *USH2A*  *USP45*  *VCAN*  *VPS13B*  *WDPCP*  *WDR19*  *WHRN*  *ZFYVE26*  *ZNF408*  *ZNF423* | *ABCD1*  *B9D1*  *B9D2*  *CEP41*  *CKAP4*  *CPLANE1*  *CTSF*  *DGKQ*  *DNAJC5*  *GJB2*  *GJB6*  *HCN1*  *KCTD7*  *KIF7*  *PDE6D*  *PEX10*  *PEX11B*  *PEX12*  *PEX13*  *PEX14*  *PEX16*  *PEX19*  *PEX26*  *PEX3*  *PEX5*  *TCTN1*  *TCTN2*  *TCTN3*  *TMEM138*  *TMEM67* | *ADIPOR1*  *AHR*  *ARMS2*  *ASRGL1*  *C2*  *C3*  *CCDC51*  *CCT2*  *CEP162*  *CEP19*  *CFB*  *CLCC1*  *CLCN2*  *CLEC3B*  *CLUAP1*  *COQ4*  *COQ5*  *COQ8B*  *DMD*  *DNM1L*  *DYNC2I2*  *ELOVL1*  *ENSA*  *ESPN*  *EXOSC2*  *FBLN5*  *HKDC1*  *HTRA1*  *IFT43*  *KIF3B*  *LRRTM4*  *MAPKAPK3*  *MIEF1*  *MMP19*  *OPN1SW*  *POC5*  *PROS1*  *RB1*  *SAMD11*  *SLC39A12*  *TBC1D32*  *TLR3*  *TLR4*  *VWA8* | *AIRE*  *ALDH3A2*  *ALPK1*  *CFAP20*  *COL18A1*  *COL4A1*  *COL9A2*  *COL9A3*  *CTC1*  *CTNNB1*  *CTNND1*  *ERCC8*  *GPR143*  *HCCS*  *IDH3A*  *IFT74*  *IKBKG*  *LAMP2*  *LRP2*  *MCOLN1*  *MED12*  *MSTO1*  *P3H2*  *POMT1*  *PYGM*  *RNU4ATAC*  *SCAPER*  *SGSH*  *SLC6A6*  *SRD5A3*  *SSBP1*  *STN1*  *TINF2*  *TMEM218*  *TRAF3IP1*  *TUBB4B*  *ZFYVE26* | *ADGRA3*  *BBIP1*  *CA4*  *CIB2*  *CISD2*  *DHX38*  *DTHD1*  *EMC1*  *FSCN2*  *GDF6*  *HARS1*  *HMCN1*  *IFT81*  *INVS*  *ITM2B*  *MFN2*  *MTRFR*  *NEK2*  *NR2F1*  *OPA1*  *OPA3*  *OR2W3*  *PDE6H*  *PDZD7*  *PGK1*  *PITPNM3*  *RDH11*  *RGS9BP*  *RIMS1*  *RTN4IP1*  *SEMA4A*  *SLC25A46*  *SLC4A7*  *SLC7A14*  *SPP2*  *TEAD1*  *TMEM126A*  *TRIM32*  *TTPA*  *WFS1*  *ZNF513* | *AMACR*  *ARL13B*  *CLN5*  *CLN6*  *CLN8*  *CTSD*  *GRN*  *MMACHC*  *PEX6*  *TMEM231*  *TPP1*  *TTC21B*  *VPS13B* | *AFG3L2*  *AGBL5*  *ARHGEF18*  *ARSG*  *ATOH7*  *ATXN7*  *CAPN5*  *CEP78*  *COL11A1*  *COL2A1*  *COL9A1*  *COQ2*  *CWC27*  *DYNC2H1*  *ERCC6*  *GNB3*  *GNPTG*  *MPDZ*  *NBAS*  *PCYT1A*  *PDSS1*  *POMGNT1*  *PRDM13*  *RCBTB1*  *REEP6*  *RIMS2*  *SAMD7*  *SLC37A3*  *SLC38A8*  *SLC66A1*  *SUMF1*  *TLCD3B*  *UBAP1L*  *USP45* | *ABCA4*  *ABCC6*  *ABHD12*  *ACBD5*  *ACO2*  *ADAM9*  *ADAMTS18*  *ADGRV1*  *AHI1*  *AIPL1*  *ALMS1*  *ARL2BP*  *ARL3*  *ARL6*  *ATF6*  *BBS1*  *BBS10*  *BBS12*  *BBS2*  *BBS4*  *BBS5*  *BBS7*  *BBS9*  *BEST1*  *C1QTNF5*  *CFAP410*  *PCARE*  *CFAP418*  *CABP4*  *CACNA1F*  *CACNA2D4*  *CC2D2A*  *CDH23*  *CDH3*  *CDHR1*  *CEP164*  *CEP250*  *CEP290*  *CERKL*  *CFH*  *CHM*  *CLN3*  *CLRN1*  *CNGA1*  *CNGA3*  *CNGB1*  *CNGB3*  *CNNM4*  *CRB1*  *CRX*  *CSPP1*  *CTNNA1*  *CYP4V2*  *DHDDS*  *DRAM2*  *EFEMP1*  *ELOVL4*  *EYS*  *FAM161A*  *FLVCR1*  *FZD4*  *GNAT1*  *GNAT2*  *GPR179*  *GRK1*  *GRM6*  *GUCA1A*  *GUCA1B*  *GUCY2D*  *HGSNAT*  *HK1*  *HMX1*  *IDH3B*  *IFT140*  *IFT172*  *IFT27*  *IMPDH1*  *IMPG1*  *IMPG2*  *INPP5E*  *IQCB1*  *JAG1*  *KCNJ13*  *KCNV2*  *KIAA1549*  *KIF11*  *KIZ*  *KLHL7*  *LAMA1*  *LCA5*  *LRAT*  *LRIT3*  *LRP5*  *LZTFL1*  *MAK*  *MERTK*  *MFRP*  *MFSD8*  *MIR204*  *MKKS*  *MKS1*  *MTTP*  *MVK*  *MYO7A*  *NDP*  *NEUROD1*  *NMNAT1*  *NPHP1*  *NPHP3*  *NPHP4*  *NR2E3*  *NRL*  *NYX*  *OAT*  *OFD1*  *OPN1LW*  *OPN1MW*  *OTX2*  *PANK2*  *PAX2*  *PCDH15*  *PDE6A*  *PDE6B*  *PDE6C*  *PDE6G*  *PEX1*  *PEX2*  *PEX7*  *PHYH*  *PLA2G5*  *PLK4*  *PNPLA6*  *POC1B*  *PPT1*  *PRCD*  *PROM1*  *PRPF3*  *PRPF31*  *PRPF4*  *PRPF6*  *PRPF8*  *PRPH2*  *PRPS1*  *RAB28*  *RAX2*  *RBP3*  *RBP4*  *RD3*  *RDH12*  *RDH5*  *RGR*  *RGS9*  *RHO*  *RLBP1*  *ROM1*  *RP1*  *RP1L1*  *RP2*  *RP9*  *RPE65*  *RPGR*  *RPGRIP1*  *RPGRIP1L*  *RS1*  *SAG*  *SDCCAG8*  *SLC24A1*  *SNRNP200*  *SPATA7*  *TIMM8A*  *TIMP3*  *TMEM216*  *TMEM237*  *TOPORS*  *TREX1*  *TRNT1*  *TRPM1*  *TSPAN12*  *TTC8*  *TTLL5*  *TUB*  *TUBGCP4*  *TUBGCP6*  *TULP1*  *UNC119*  *USH1C*  *USH1G*  *USH2A*  *VCAN*  *WDPCP*  *WDR19*  *WHRN*  *ZNF408*  *ZNF423* |
